# Testing the Acceptability and Feasibility of a Gender-Informed Smoking Cessation mHealth App for Women: Mixed Methods Approach

**DOI:** 10.2196/71683

**Published:** 2025-09-25

**Authors:** Osnat C Melamed, Kamna Mehra, Allison Gayapersad, Roshni Panda, Nadia Minian, Laurie Zawertailo, Leslie Buckley, Marta Maslej, Lorraine Greaves, Andreea C Brabete, Jonathan Rose, Matt Ratto, Peter Selby

**Affiliations:** 1 INTREPID Lab, Nicotine Dependence Service, Centre for Addiction and Mental Health, 1025 Queen Street West, Toronto, ON, M6J1H4, Canada, 1 416 535 8501; 2 Department of Family and Community Medicine, University of Toronto, Toronto, ON, Canada; 3 Campbell Family Mental Health Research Institute, Centre for Addiction and Mental Health, Toronto, ON, Canada; 4 Institute of Medical Sciences, University of Toronto, Toronto, ON, Canada; 5 Department of Pharmacology and Toxicology, University of Toronto, Toronto, ON, Canada; 6 Addictions Division, Centre for Addiction and Mental Health, Toronto, ON, Canada; 7 Department of Psychiatry, University of Toronto, Toronto, ON, Canada; 8 Krembil Centre for Neuroinformatics, Centre for Addiction and Mental Health, Toronto, ON, Canada; 9 Centre of Excellence for Women's Health, Vancouver, BC, Canada; 10 School of Population and Public Health, Faculty of Medicine, University of British Columbia, Vancouver, Canada; 11 Edward S. Rogers Sr. Department of Electrical and Computer Engineering, University of Toronto, Toronto, Canada; 12 Faculty of Information, University of Toronto, Toronto, Canada; 13 Schwartz Reisman Institute for Technology and Society, University of Toronto, Toronto, Canada; 14 Dalla Lana School of Public Health, University of Toronto, Toronto, Canada

**Keywords:** women’s health, gender, smoking cessation, mHealth, digital health, co-development, feasibility, mobile phone, mobile health

## Abstract

**Background:**

Cigarette smoking is a leading cause of preventable morbidity and mortality worldwide. Women who smoke face greater health risks than men, including higher rates of cardiovascular disease and more pronounced declines in lung function. Despite this, women experience lower success rates with conventional smoking cessation treatments, due in part to unique sex- and gender-related factors influencing smoking behavior and barriers to quitting. Digital health tools, such as mobile health apps, offer a promising avenue for delivering accessible, tailored smoking cessation support to women.

**Objective:**

This study evaluated the acceptability and feasibility of the “My Change Plan–Women” (MCP-W) app, a gender-specific smoking cessation mobile health intervention co-designed with women who smoke, clinicians, and researchers, to address women’s unique needs in smoking cessation.

**Methods:**

We conducted a single-group, prospective, sequential mixed methods study with 30 women who smoke in Ontario, Canada. Participants used the MCP-W app for 28 days. Acceptability was defined as ≥50% of participants endorsing “agree” or “strongly agree” to the statement “using the app is likely to help me make changes to my smoking habits.” Feasibility was defined as ≥50% of participants using the app for 7 or more days during the trial period. Quantitative data on acceptability, smoking behavior, and motivation to quit were collected at baseline and follow-up via REDCap (Research Electronic Data Capture) surveys. App usage metrics were captured through Google Analytics. Semistructured interviews explored participants’ experiences using the app and were thematically analyzed using the theoretical framework of acceptability.

**Results:**

At follow-up, 37% (11/30; 95% CI 21%-56%) of participants rated the MCP-W app as acceptable, falling below the predefined threshold (≥50%) and indicating that the intervention “needs further work.” Feasibility criteria were met, with 60% (18/30) of participants using the app for 7 or more days. Notably, acceptability was higher among those who used the app for more than 14 days (7/11, 64%) compared with those with lower usage (4/19, 21%). Average daily cigarette consumption decreased from 16.4 to 14.6 cigarettes, and the number of participants reporting at least 1 smoke-free day in the previous week increased from 7% (2/27) to 22% (6/27). Qualitative findings revealed that women with higher motivation to quit found the app more helpful, particularly its behavior change tools (eg, cigarette tracking and identifying triggers) and gender-specific content. However, women facing stress, mental health challenges, or low readiness found it harder to engage. Participants suggested enhancements including customizable reminders, more interactive content, and live or artificial intelligence–based emotional support.

**Conclusions:**

The MCP-W app is a feasible intervention for delivering gender-specific smoking cessation support. However, its acceptability was limited to a third of users with high levels of motivation. Improvements to interactivity and support features may enhance its relevance and uptake among women with complex barriers to quitting.

## Introduction

Cigarette smoking is one of the leading risk factors of morbidity and mortality globally contributing to the development of cancers, lung diseases, and heart diseases [1]. While the prevalence of smoking among women in some Western countries may be reducing overall, the rate of decline is slowing, and the contribution of smoking to deaths among women is increasing [2,3]. Smoking causes more harm in women compared with men. Relative to men, women who smoke have higher rates of cardiovascular events and experience greater decline in lung function as a result of smoking [4]. When trying to quit smoking to protect their health, women experience less success than men when offered evidence-based smoking cessation treatment. The disparity can be partly attributed to sex- and gender-related factors affecting motives for smoking and barriers to quitting, which are rarely addressed in treatment. This highlights the need for smoking cessation programs to take into account the unique needs of women and incorporate them into treatment.

Various sex- and gender-related factors affect smoking and quitting behaviors among women. Cosgrove et al [5] found differences between the effect of nicotine on the dopaminergic pathway in the brains of men and women, where men showed a greater dopamine response to smoking than women. This suggests that men smoke for the pleasurable effects of nicotine, whereas smoking has additional drivers in women. Others theorized that stressful life experiences in women become a conditioned cue for smoking (ie, sociopharmacology) [6]. This theory is backed by evidence of high smoking rates among women facing material hardship, domestic violence, mental illness, and lone parenting. Biological factors, as well as diverse social and psychological factors (ie, sex- and gender-related factors), contribute to a greater difficulty in smoking cessation among women relative to men [4,7-11]. Previous work suggests a number of unique challenges experienced by women in quitting, including using smoking to cope with stress or manage emotions and control weight by suppressing their appetite [12]. Behavioral interventions that focus explicitly on the unique experiences of women trying to quit could improve smoking cessation success [11].

Mobile health (mHealth) technology, such as smartphone apps, can deliver smoking cessation treatment to wider populations in a more cost-effective manner [12-16]. For greater effectiveness, mobile app content can be tailored to address the unique smoking cessation needs of women. Indeed, 4 apps dedicated to helping women reduce or quit smoking have been identified in a systematic review of the literature [13]; 2 of them focus on women who are pregnant [17,18], 1 on modifying multiple health behaviors among women [19], and 1 for Chilean women using which sends motivational messages and strategies to women to reduce or quit smoking [20]. This trend is encouraging, but there is room for further work focused on a wider, more general population of women who smoke.

The “My Change Plan–Women” (MCP-W) app is one such additional mHealth intervention. The original version of the app, My Change Plan (MCP), was developed to support the smoking cessation efforts of patients enrolled in the Smoking Treatment for Ontario Patients (STOP) program and was a digital evolution of a paper-based booklet, also called “My Change Plan” [21,22]. The MCP app provides evidence-based behavioral change techniques to support tobacco reduction and cessation that can be helpful for both women and men in developing their quit plan. The quit plan includes goal setting, identifying smoking triggers, and developing coping strategies which are grounded in theory [23-25]. It allows users to track their cigarettes and cravings and evaluate financial savings from reducing or quitting smoking. It also provides evidence-based information in the app’s library about smoking cessation medications, local supports for smoking cessation (eg, smoker’s helpline), and the impact of physical activity, nutrition, stress, and sleep on smoking and smoking cessation. The MCP app has been available for iPhone (Apple Inc) and Android (Google) smartphones, in their respective app stores, for over 5 years. The MCP-W app retains MCP gender-neutral content and complements it with women-specific content that focuses on women’s unique motives for smoking and the barriers to quitting they experience. The women-specific content is delivered through 4 animation videos embedded in the app, and testimonials from women who smoked are included in the app’s library. The animated videos focus on women-specific motives for smoking, challenges faced by women when trying to quit, and strategies that have helped women overcome them. The testimonials include quotes from women on topics such as “desire to quit,” “quit strategies,” “quit struggles,” and “encouragement.”

The women-specific version of this app, the MCP-W app, was co-designed in collaboration with women who smoke (ie, patient partners) and a multidisciplinary team of researchers and clinicians with expertise in tobacco addiction, implementation science, sex and gender science, women’s health, public health, and app development [26]. The MCP-W app was based on motivational interviewing. This is a counseling method aimed to promote behavioral change in a compassionate and nonjudgmental manner [27]. It respects women’s autonomy to make changes to their smoking at their own pace, when they feel ready, and does not pressure them into quitting by using scare or shame tactics.

The MCP-W app was developed between July 2022 and August 2023, and a testable version was finalized in January 2024. The aim of this study is to test whether women who smoke find the MCP-W app to be an acceptable tool for the delivery of gender-informed smoking cessation support, and whether the MCP-W app can feasibly deliver this content.

## Methods

### Study Design and Setting

We conducted a single-group, prospective, sequential mixed methods study to test the acceptability and feasibility of the MCP-W app at the Center for Addiction and Mental Health (CAMH) INTREPID Lab in Toronto, Ontario, Canada. A comprehensive protocol of this study was published previously [26].

### Participant Recruitment and Study Procedures

Purposive sampling was conducted between February and May 2024 to recruit 30 women participants who smoked cigarettes to test the MCP-W app. The study recruitment flyer was shared through monthly presentations to STOP clinicians, who provide smoking cessation treatment in primary care clinics across Ontario, Canada. In addition, recruitment flyers were provided to health care practitioners at the Nicotine Dependence Clinic at CAMH and posted on the CAMH research boards across campus. Furthermore, past treatment-seeking individuals (STOP program participants), who agreed to participate in future research studies, were contacted about the study.

Women who expressed interest in participation were screened for eligibility over the telephone. We included women between 25 and 65 years of age, who smoked at least 5 tobacco cigarettes per day, owned a smartphone, had continuous internet access, and had experience using smartphone apps. Those who did not feel comfortable using smartphone apps, or were pregnant, breastfeeding, or trying to become pregnant in the next 3 months were ineligible to participate. Individuals with severe general or mental health conditions that could interfere with study participation were also excluded. This criterion was assessed during the eligibility screening and applied to those unable to complete study procedures due to a physical or mental illness (eg, current hospitalization). Eligible participants provided informed consent over WebEx (Cisco Systems), a secure online platform to conduct videoconference meetings, after a discussion of the study procedures, risks, benefits, and rights as a study participant. During this WebEx meeting, participants also completed the baseline survey (MultimediaAppendices 1  2) through a unique REDCap (Research Electronic Data Capture) survey link sent by email, where they provided information about their sociodemographic characteristics, acceptability of the app, smoking behaviors, smoke-free days in the past week, and their motivation to quit smoking. They downloaded the MCP-W app (either an Android or an iPhone version) and were provided a brief demonstration of the various app features.

Participants were granted access to the app for a period of 28 days. After the 28-day trial completion, participants were sent an automatic email through REDCap with a follow-up survey. The follow-up survey asked participants about the acceptability of the app, smoking behaviors, smoke-free days in the past week, motivation to quit smoking, the usability of the app using the System Usability Scale (SUS) [28], and how likely they were to recommend the app to others (MultimediaAppendices 2  3). In addition, participants were invited to schedule the follow-up semistructured WebEx interview about their experiences using the app. Interviews were conducted by a research staff member who did not have any previous relationship with the participants. The interview guide consisted of questions about the app based on the 7 constructs of the theoretical framework of acceptability (TFA; ie, affective attitude, burden, ethicality, intervention coherence, opportunity costs, perceived effectiveness, and self-efficacy) [29], as well as additional questions about barriers to quitting smoking, the app’s helpfulness in quitting smoking, and strategies to make the app helpful for women from diverse backgrounds (Multimedia Appendix 4). The interview lasted for an average of 34 (SD 11, range 18‐62) minutes. The interviews were automatically transcribed by the WebEx Assistant Software. Research staff members reviewed, cleaned, and verified the transcripts for accuracy.

### Data Analysis

#### Primary Outcomes

The primary outcomes for the study included acceptability and feasibility of the app. Acceptability of the app was measured by asking the question “using the app is likely to help me make changes to my smoking habits” at baseline and follow-up surveys, which could be answered on a 5-point Likert scale from “strongly agree” to “strongly disagree.” The proportion of participants endorsing “agree” or “strongly agree” was calculated. The app would be considered “acceptable” if the majority (≥50%) of participants rate it as such. Similarly, if 26% to 50% reported the app as acceptable, then it would be deemed “needs further work”; otherwise, it would be deemed “unacceptable” if the proportion was 25% or lower. Nonresponse values were imputed to “do not agree” since low study participation most likely indicated low acceptability of the app.

Feasibility of the app was assessed using Google Analytics data. Participants were assigned unique log-in IDs and the number of times they accessed the app and clicked on the videos was collected using Google Analytics. The app would be considered “feasible” if the proportion of participants who accessed the app for 7 days or more out of 28 days was 50% or more, “needs further work” if 26% to 49% , and “not feasible” if 25% or less. Since the Google Analytics data was available for all participants, there was no missing data on feasibility measures. The binomial 95% CIs were calculated to assess precision of estimates, but the decision about acceptability and feasibility rested on point estimates.

#### Secondary and Tertiary Outcomes

The secondary outcome measures consisted of participants’ motivation to quit smoking. Changes in mean scores of motivation from baseline to follow-up were calculated for each of the 3 validated subscales: “importance,” “commitment,” and “ability” [30]. Participants rated the following statements: (1) it is important for me to change my smoking habits, (2) I am trying to change my smoking habits, and (3) I could change my smoking habits, using a Likert scale where 1=definitely not, 2=probably not, 3=maybe, 4=probably, and 5=definitely.

The tertiary outcomes consisted of smoking behavior metrics including change in the number of cigarettes smoked daily and the number of smoke-free days in the last 7 days. Quantitative data was analyzed using Stata v.16 (StataCorp). Based on the observed distribution, we used McNemar tests for binary outcomes, exact Wilcoxon signed-rank tests for paired ordinal data, and negative binomial regression for cigarettes per day. Demographic data were analyzed descriptively.

#### Qualitative Data Analysis

A deductive analysis approach was used to analyze the qualitative data [31]. Using Microsoft Excel, the interview data elicited from the 26 participants were organized into categories based on the 7 TFA constructs and 2 additional questions asked in the interview guide. KM read and reread all transcripts to become familiar with and code the data. AG and OM reviewed the transcripts and the extracted and coded data to ensure accuracy and consistency. KM, OM, and AG met weekly to discuss and refine the codes. Coded data were structured into themes and relevant quotes were selected to support themes and subthemes.

### Ethical Considerations

The study was approved by the CAMH Research Ethics Board (#122/2023). Participants provided informed consent via WebEx (Cisco Systems). Participants were provided an honorarium of CAD $30 (a currency exchange rate of US $1=CAD $1.33796 was applicable at the time of data collection) for completing the baseline survey and an additional CAD $30 for completing the posttrial interview. No identifying information about the participants has been included in this manuscript.

## Results

### Characteristics of Study Participants

A total of 383 women were contacted about the study (369 from the STOP program and 16 from other sources), out of which 55 women expressed interest and were screened for eligibility. Of the total, 49 women were found to be eligible, out of which 32 women provided informed consent and completed the baseline survey. Furthermore, 30 women were able to download the app, and 2 women could not do so due to technical issues, 27 completed the follow-up survey, and 26 completed the interview (3 participants were lost to follow-up). The baseline demographic data of the 30 participants are described in Table 1.

**Table 1.** Characteristics of 30 study participants.

Demographic characteristics	Value
Age (y), mean (SD)	49.5 (9.4)
Gender, n (%)	
Woman	30 (100)
Marital status^a^, n (%)	
Never legally married	10 (33)
Legally married (and not separated)	10 (33)
Separated or divorced	4 (13)
Living with a partner but not married (common law)	5 (17)
Highest level of education completed, n (%)	
Some high school	1 (3)
High-school diploma	5 (17)
Some college	5 (17)
College diploma	13 (43)
University degree	6 (20)
Total household income last year before taxes (CAD $^b^), n (%)	
40,000 or less	8 (27)
40,000 to 80,000	12 (40)
More than 80,000	10 (33)
Racial background, n (%)	
Métis	2 (7)
Latin American (Hispanic or Latin American descent)	1 (3)
Middle Eastern (eg, Arab, Persian, and West Asian descent [Afghan, Egyptian, Iranian, Kurdish, Lebanese, Turkish, etc])	1 (3)
South Asian (eg, Bangladeshi, Indian, Indo-Caribbean, Pakistani, and Sri Lankan descent)	1 (3)
White (eg, European descent)	25 (83)
Overall health, n (%)	
Excellent	0 (0)
Very good	5 (17)
Good	15 (50)
Fair	9 (30)
Poor	1 (3)
Chronic health conditions, n (%)	
High blood pressure	7 (23)
High cholesterol	8 (27)
Heart disease	2 (7)
Diabetes	6 (20)
Chronic bronchitis, emphysema, or COPD^c^	3 (10)
Rheumatoid arthritis	3 (10)
Chronic pain	11 (37)
Cancer	1 (3)
Depression	11 (37)
Anxiety	13 (43)
Schizophrenia	1 (3)
Substance use disorder (other than tobacco or caffeine)	2 (7)
Problem gambling	1 (3)
Cigarettes smoked per day, mean (SD)	16.4 (7.5)

a One participant selected “Don’t know/prefer not to answer.”

b Data collected in Canadian dollars. A currency exchange rate of US $1=CAD $1.33796 was applicable at the time of data collection.

c COPD: chronic obstructive pulmonary disease.

### Primary Outcome Measures

#### Acceptability of the MCP-W App

Following the 28-day trial period, participants responded to the following question: “Please indicate how much you agree or disagree with the following statement: Using the app is likely to help me make changes to my smoking habits.” In total, 11 out of 30 (37%, 95% CI 21%-56%) participants endorsed “agree or strongly agree.” This result did not reach our predefined threshold for acceptability (≥50%), but landed in the “needs further work” range (26%‐49%). We assessed the acceptability of the intervention before and following the 28-day trial, in which the proportion of participants endorsing “agree or strongly agree” decreased from 43% (13/30) before using the app to 37% (11/30) at follow-up; however, this decrease was not statistically significant (Table 2).

**Table 2.** Primary, secondary, and tertiary outcome measures.

Outcome measures	Baseline	Follow-up	*P* value
Primary outcome			
Acceptability^a^, n (%)			.09
Agree or strongly agree	13 (43)	11 (37)	
Neutral, disagree, or strongly disagree	14 (47)	16 (53)	
Secondary outcome			
Motivation^b^, mean (SD)			
Importance	4.7 (0.6)	4.7 (0.6)	>.99
Commitment	4.6 (0.7)	4.5 (0.7)	.22
Ability	4.1 (0.9)	3.7 (1.0)	.40
Tertiary outcomes			
Cigarettes smoked per day^b^, mean (SD)	16.4 (7.5)	14.6 (7.0)	.01
1 or more smoke-free days in the last 7 days^b^, n (%)	2 (7)	6 (22)	.01

a Acceptability was measured on a 5-point Likert scale using the question: “Using the app is likely to help me make changes to my smoking habits.”

b Data from 27 participants who completed the follow up survey.

#### Feasibility of the MCP-W App

Based on the Google Analytics data, 18 (60%, 95% CI 41%-77%) participants used the app for 7 or more days, which fulfilled the 50% feasibility criteria. Furthermore, 14 (47%) participants used the app for 1‐7 days, 5 (17%) used it for 8‐14 days, 3 (10%) for 15‐21 days, and 8 (27%) for 22‐28 days. Based on the number of days the MCP-W app was used, the proportion of participants who found the app acceptable was 14% (2/14; 1‐7 days), 40% (2/5; 8‐14 days), 67% (1/3; 15‐22 days), and 63% (3/8; 22‐28 days; Multimedia Appendix 5). There was a statistically significant correlation between the number of days the app was used and agreement with the acceptability of the app (*P*<.001).

### Secondary and Tertiary Outcome Measures

#### Motivation to Quit

Participants had a high level of motivation with respect to “importance” of quitting smoking (mean score of 4.7, SD 0.6), and it did not significantly change from baseline to follow-up. There were no statistically significant changes in the “commitment” and “ability” measures (Table 2).

#### Smoking Behaviors

The number of cigarettes smoked per day significantly reduced from a mean of 16.4 (SD 7.5) cigarettes at baseline to a mean of 14.6 (SD 7.5) cigarettes at follow-up. The number of participants endorsing 1 or more smoke-free days in the last 7 days significantly increased from 2 (7%) at baseline to 6 (22%) at follow-up (Table 2). Based on the number of days the MCP-W app was used, the proportion of participants who reported a smoke-free day in the last 7 days at follow-up was 0% (1‐7 days), 20% (8‐14 days), 100% (15‐22 days), and 25% (22‐28 days). Furthermore, 1 participant reported quitting smoking at follow-up.

#### System Usability

The mean total score of the SUS was 70.2 (SD 12). The mean score of the response for the question “How likely are you to recommend this app to others?” was 6 (SD 2.0; on a scale of 0 to 10).

### Qualitative Inquiry Into the Acceptability and Feasibility of the MCP-W App

Table 3 summarizes key themes and insights from qualitative inquiry with MCP-W participants.

**Table 3.** Summary of key themes and insights from qualitative results.

Theme	Subthemes or key insights	Supporting quotes
Motivation and readiness to quit	App more useful for those highly motivated or ready to quit Lower readiness linked to lower engagement	“I was pretty motivated... that gave me a definitive start date.” [ID 27] “I’m not there yet... the app helped …but I think you have to be a 100% ready.” [ID 01]
Barriers to quitting smoking	Stress, mental health, daily hardships Habitual use and nicotine addiction Social contexts and emotional triggers	“The more stressed I am, the more I smoke...” [ID 19] “It’s really hard to quit... the brain always makes excuses to get it” [ID 28]
Helpful app features	Cigarette tracking increased awareness Trigger identification and coping strategies Resource library, visual feedback (savings, graphs, progress) Gender-specific content resonated with users	“...just reminding me to get outside and…do something other than just …smoking...” [ID 11] “Everything they were talking about (in the videos) is how I felt...” [ID 18]
Features in need of improvement	Burden of frequent tracking/logging Usability issues (eg, journal entries, notifications) Desire for more interactivity and real-life testimonials	“It just seemed like the app wanted me to go into it every time...” [ID 07] “I would like to get more like a free open option to write stuff down” [ID 19]
Desire for additional support	Suggestions for live support or artificial intelligence–based chat features App more effective when paired with counseling or pharmacotherapy	“A live connection... someone like ...a sponsor.” [ID 14] “Maybe they should be starting that (medication) already before they use the app, so they already have a jump start, so to speak” [ID 29]

#### Motivation and Readiness to Quit Smoking

We found that participants’ motivation and readiness to quit smoking influenced the acceptability and feasibility of the MCP-W app. More than half (n=16) of all participants interviewed mentioned that they were motivated to quit smoking. The app was seen as a helpful tool particularly for those highly motivated to quit. Some doubted the app’s utility for women with lower levels of motivation or readiness.

*Well, right now for my journey, I’m at a point where I know - I know I’ve got to quit smoking for my health, for my family’s health, but mainly for mine just because I’m getting older I smoked for so long. Um, here’s my cough…*

[ID 03]

*Well, I was pretty motivated when you first contacted me, so I think that’s what helped [quit] was with the trial starting that that gave me a definitive start date or an end date, I should say.*

[ID 27]

*And I’m so tired of smoking, I don’t want to smoke anymore. But I’m not there yet. I don’t think, cause, I mean, I could analyze my smoking and I could, you know, the app helped me with that and, and figure it out and the triggers and… But, I think you have to be a 100% ready.*

[ID 01]

Participants who mentioned that they were motivated and ready to quit smoking appeared to find the MCP-W app feasible, which they demonstrated by engaging with the MCP-W app longer than participants who mentioned they were not as motivated or ready. For example, one of the following participants who was ready to quit used the MCP-W app for 29 days compared with the other participant who was not ready and only used the MCP-W app for 14 days.

*But I’m determined right now with this app… It like with the full 29 days I’ve been using the app so far…*

[ID 31]

*I feel like, I have no motivation or willpower or something, and I’m just not making real changes here. So this is just kind of like, maybe it’ll help other women, but not me.*

[ID 16]

Some participants felt that the app is of most use to women who are ready to quit. Since they did not feel ready, it was less relevant to their needs.

*If I was further into this stage of quitting smoking or wanting to quit smoking, it would definitely - it would help me. I just - I’m not there yet, so it was easy for me to not-not use the app, to not look at it. Right?*

[ID 03]

*I think that if somebody is ready, that it would be a valuable tool. It would be like an accountability tool… So I think I would be a good candidate for that if and when I was ready, I was just not ready…*

[ID 32]

Participants cited numerous barriers to quitting smoking, some related to gender and others to the nature of the tobacco addiction including daily stressors (eg, employment, housing, finance, and relationships; n=16), the addictive nature of nicotine (eg, lifelong habit and hard to give up; n=14), boredom or nonstructured daily routine (n=4), social smoking (eg, with partners, friends, and work colleagues or managers; n=3), and mental health challenges (eg, depression; n=3).

*The more stressed I am, the more I smoke, so, stress is a big trigger. I think when you’re looking at, like, certainly me being a single mom, everything’s on me to do all the time. So, I think quitting smoking would be a lot easier if I wasn’t so stressed out all the time.*

[ID 19]

*I think that I would not have to love nicotine. Or I think that I would not have ever had to start smoking… But personally, I just don’t think, once a person starts and once their brain like becomes addicted to nicotine, it’s, it’s really hard to quit cause the brain always makes excuses to get it.*

[ID 28]

Some participants mentioned that the tailored content in the app was helpful to overcome gender-specific barriers to quitting.

*I was able to cut out a few cigarettes when I’m walking to the bus stop. I made a conscious effort, because I thought, oh, that is what I really could skip. Um, most of my problem is that it is habit. I think the reasons why we smoke and when we’re smoking, when you see it [in the MCP-W app], it really hits home. It’s like, wow.*

[ID 02]

*I think since I’ve started using this [MCP-W app], I have reduced. So proud of that, um. It’s just a matter of reducing to nothing.*

[ID 10]

*So, it actually made me think about that in retrospect of where I’ve been and what I’ve been doing and even just reminding me to get outside and go and do something other than just sitting here smoking. Just seeing that sheer number and volume day by day, helped me cut back. By the end of the month, it helped me cut back probably 10 to 11 cigarettes per day.*

[ID 11]

#### MCP-W App Features That Were Helpful

Participants provided additional insights about the acceptability and feasibility of the MCP-W app. Factors that facilitated the acceptability and feasibility of the MCP-W app included the available tools and resources, for example, the feature of tracking the number of cigarettes they smoked. Participants (n=15) mention this feature helped them become more aware of the number of cigarettes they were smoking and held them accountable for changing their behavior (n=6). When tracking their cigarettes, they also became more aware of their smoking triggers such as emotions, places, and people they were with (n=10).

*I like the idea that when I did use it, that it would ask me my, my attitude or who I was smoking with, which then if I used it properly, then it would trigger to when I am actually smoking. And then it would make me more aware of where I need to maybe change my surroundings or the urge to smoke.*

[ID 04]

Participants mentioned that identifying their triggers and recording their coping strategies was helpful (n=15).

*I did like the survey where you could put your motivations for quitting, how many you smoked a day, what to do, what are your triggers, how you could alleviate those stresses… when you think about it beforehand, so that when you’re in the moment, you’re able to go back to that app, look at it and say, “Okay, I said I would do this.*”

[ID 23]

Participants thought that the library feature provided helpful information about resources on quitting smoking, medications, such as nicotine replacement therapy, managing weight when quitting, and so on (n=14).

*The library section had quite a bit of information as well with different smoking cessation medications and different supports and things like that that are very helpful.*

[ID 25]

Other features found helpful by participants included visualizing their smoking through progress graphs (n=7), receiving notifications about reduction in number of cigarettes (n=6), and feedback about money saved (n=4).

*I liked the notifications. So, when it said to me, like, you’ve smoked, you know, so many cigarettes less this morning than you did yesterday, that was kind of like a pat in the back moment for me.*

[ID 01]

A number of participants provided feedback on the gender-specific content embedded in the app. In total, 9 participants mentioned the topics depicted in the videos and testimonials tailored to women who smoke resonated with them and 5 mentioned that the videos motivated them or provided them information they did not know.

*You know, I was reading the testimonials of people and it’s, it’s real people and you, you know, you can relate to them and then it just gives you more motivation to want to quit smoking.*

[ID 04]

*I think every video hit home, because everything they were talking about is how I felt or how I feel, right? So it kind of, that reminded me of like my own personal journey as well. So there was a lot of things in those videos that was just totally me.*

[ID 18]

#### MCP-W App Features That Needed Improvement

Using the app requires a time commitment which was perceived as a burden to some. Half the participants (n=13) mentioned they found it difficult to find time or forgot to track every cigarette they smoked, and several (n=12) mentioned they needed reminders to log their cigarettes, with some specifying the need for customizable reminders 2 or 3 times a day. Indeed, 5 participants mentioned that women with children would have difficulty logging their cigarettes multiple times a day every day. Furthermore, 4 participants also reported logging factors associated with every cigarette was too tedious.

*It just seemed like the app wanted me to go into it every time I had a cigarette and I just don’t have that kind of time or resources to do that, especially while I’m at work - and like if you’ve got a young mom or anything like that, there’s no way they could do that. Like, it’d be better if we could go in a couple of times a day and just pinpoint on a timeline when we had cigarettes.*

[ID 07]

Some participants described functionality and design issues with key features of the MCP-W app that may have affected the usability. For example, some participants mentioned the journal did not allow independent entries (n=7), clicking on notifications did not open the app (n=2), or logging previous cigarettes did not allow logging the factors associated with them (n=4).

*My journal part… I couldn’t enter anything… I think it would be really beneficial if we can write things like ‘Today really sucks and I’m really struggling with quitting smoking”… I would like to get more like a free open option to write stuff down.*

[ID 19]

*Sometimes I noticed on my phone, I’d get a notification pop up. And then I’d click on it, but it never said anything. So, I don’t know if there were some problems with that.*

[ID 10]

A few participants (n=5) wished for the MCP-W app to be more engaging in order to maintain participants’ interest over time. Participants suggested that the MCP-W app should include interactive activities (ie, gamification) or dynamic content to keep interest levels high (n=8).

*Maybe that’s a good time to have something integrated into the app, like a game to play…*

[ID 19]

*Because there was not a true engagement. At the end of the day an app, it passes the excitement like a movie or like a show, like anything. It passes the time. So if there’s no more like new things or like more engagement you will lose just interest naturally, I think…*

[ID 22]

In total, 15 participants acknowledged that the app contains both gender-specific and gender-neutral content. Furthermore, 2 suggested that the library could be improved by including more gender-related information such as hormonal influences on smoking. They also preferred human actors in the videos as opposed to the animated characters (n=9), with additional success stories and strategies to overcome challenges to quit (n=6).

*I think having a section to deal with hormones would be very beneficial like… things have changed drastically for me there, going through menopause… So, I think having that information involved, so you can take a closer look at yourself, because hormones trigger everything in your life, really.*

[ID 11]

*Oh, maybe, like real people (in the videos) would be nice and also maybe people who have been successful in what worked the best for them.*

[ID 28]

A few participants (n=6) mentioned that they would like to have a feature that allows the user to connect to a support person or artificial intelligence (AI)–based chatbot for more personalized support.

*A live connection where you can phone somebody and speak to them live. And not for psychological, like, not a psychiatrist or psychologist, but just someone, like, what do they call-a sponsor?*

[ID 14]

*I think in terms of quitting and smoking, you need more live support, that’s one. But like I mentioned before, I think if it had like a chat open or linked to other participants that shared the experience, like, “Okay, how are you doing this day.*”

[ID 22]

Overall, participants (n=5) mentioned that the app can be a helpful tool, particularly when it is combined with other types of support such as behavioral counseling or smoking cessation medication, rather than a stand-alone solution.

*Well, it did [help] on the few days that I had, for whatever reason, fewer smokes than other days. I think it did tell me like something like “You didn’t have a cigarette this morning”… I think I’m stuck in some personal issues right now that that make it hard. I think you have to already be committed to doing something seriously and not have a lot of extraneous problems that are gonna defeat you before you even get started. Maybe somebody should already be starting patches or have a bunch of the other, you know, whether it’s that medication, you know, pills or whatever they are, or other things. Maybe they should be starting that already before they use the app, so they already have a jump start, so to speak. Otherwise, just jumping into the app, it’s really hard to just change everything without more, more of a push.*

[ID 29]

In summary, participants’ motivation and readiness to quit smoking significantly influenced the acceptability and feasibility of the MCP-W app. Women motivated to quit found the app helpful for tracking smoking behaviors, identifying triggers, and providing gender-specific resources. However, individuals with lower readiness to quit engaged less with the app. Participants recommended improvements, such as providing additional success stories of women, enhanced interactivity (eg, gamification), and personal coaching options. While the app could be beneficial, especially when paired with other cessation tools, it was not perceived as a stand-alone solution, particularly for individuals who face significant barriers to quitting.

## Discussion

### Principal Findings

In this study, we aimed to test the acceptability and feasibility of a gender-specific app for smoking cessation among women who smoke using a mixed method approach. Our study found that the MCP-W app was acceptable to slightly more than a third of women, with 37% (11/30) endorsing it as a helpful tool that can support their smoking cessation needs. As such, it did not meet the a priori acceptability criteria (>50%). Nonetheless, the app met feasibility criteria, with 60% (18/30) of participants using it for 7 or more days. The finding that the MCP-W app did not meet the predefined threshold for acceptability (only 11/30, 37% of participants rated the app as likely to help with smoking cessation) has important implications for future scalability and deployment. This result suggests that without additional support or enhancements, the MCP-W app in its current form may not be suitable as a stand-alone tool for a broad population of women who smoke. Instead, the app may be most useful for a subset of women who are already highly motivated to quit. This emphasizes the need for further development work to improve engagement and relevance, particularly for women exhibiting ambivalence about quitting. Future iterations of the app may benefit from incorporating more dynamic features (eg, personalized reminders, gamification, or peer support options) and tailoring content based on users’ readiness to change.

The proportion of participants needed to consider the app acceptable (50%) was not met; however, the proportion of participants who used the app more frequently (more than 14 d) found it acceptable (7/11, 64%), as compared with the proportion of participants (4/19, 21%) who found it acceptable but used the app less frequently (14 d or less). The significant correlation between acceptability and feasibility of an app could be bidirectional, representing the increased acceptability of the app resulting in more use or conversely, as users engage with the app for longer, they find it more helpful. Previous research has found a similar correlation between acceptability and feasibility, even though they have been recognized as separate constructs [32,33]. In addition, the proportion of participants who mentioned they would find the MCP-W app acceptable at baseline was 43% (13/30), demonstrating their low pre-existing beliefs about the app’s ability to help them make changes to their smoking. Research has shown that pre-existing beliefs about effectiveness can affect engagement with digital health interventions, for example, perceiving technology as distracting is linked with lower use metrics [34]. There may be a need for more education and awareness about the potential helpfulness of digital health interventions among women who are trying to reduce or quit smoking.

MCP-W use was associated with a positive change in participant smoking behaviors. The average number of daily cigarettes reduced from 16.4 to 14.6, and more women reported that they attempted to quit after using the MCP-W compared with baseline (6 vs 2). The latter was more pronounced in women who used the app more versus less than 14 days. This is an encouraging finding, although it should be interpreted with caution given that our study did not have a control group. This finding is consistent with other reports from digital interventions for smoking cessation [13,35] that speak to the potential effectiveness of digital health tools to support individuals in quitting. Our sample included women with a high level of motivation for quitting, which did not change significantly across the study timeline. Women rated the importance of quitting smoking as 4.7/5 at both baseline and follow-up. However, their perceived “ability” to quit smoking, measured by the statement “I could change my smoking habits (reduce or quit)” decreased in a nonsignificant way from 4.1/5 to 3.7/6. Since many women reported tracking their cigarettes and reducing during the trial, it is possible that these actions crystalized the challenges they face in their day-to-day lives, thus rating their ability to quit as lower.

Women’s attitudes toward quitting were closely linked with whether they rated the MCP-W app as acceptable and the relative intensity of their use of the app (feasibility). Despite the majority of women reporting high motivation for quitting for common reasons, such as improving one’s health, saving money, and for their families or children, many felt they were not ready to make this change. Lack of readiness was centered around life stressors such as material hardship, interpersonal conflict, lone parenting, mental illness, and medical problems. Dealing with the above was prioritized, and quitting smoking was left to be dealt with in the future, when life circumstances would change. This notion is consistent with the previous literature on women and smoking [7]. This explains, in part, why some women did not find the app acceptable. The app cannot directly change difficult life circumstances but is aimed at strengthening women’s resilience in light of them. It is possible that combining the use of the app with support from cessation counselors could provide women with clarity regarding the individual barriers they face and guide them to develop helpful strategies to overcome those barriers [36].

The MCP-W app contained evidence-based gender-neutral behavior change techniques meant to facilitate change via goal setting, tracking daily cigarettes, and identifying triggers for smoking [23]. Most women found these techniques helpful. This is consistent with previous research on effective features that are recommended for use in smoking cessation apps [13]. However, in our study, women who were not ready to quit tended to put less effort into these behavioral tasks (ie, logging smoked cigarettes and identifying smoking triggers), compared with women who were ready to quit. Some women also commented on the efforts needed to enter information into the app (ie, goal setting and tracking), and felt it was a big task, particularly from women with very little free time, like single mothers. It is possible that newer technologies will be able to make these tasks less tedious and easier to perform. For example, voice to app features, where you do not need to enter information manually to your device but use a voice command to do so (eg, “MCP-W, record I just smoked one cigarette in my car as I am driving back from work”). Voice-activated app functions are widely used today [37] and should be explored in the future as a method to reduce the burden of tracking a target behavior.

The MCP-W app contained gender-specific components including 4 animation videos and testimonials from women who quit smoking. The majority of women appreciated the tailored content and felt they could relate to the challenges expressed by other women. They sense that others sharing your struggles and understanding you is considered a key tenet of peer-supported activities in addiction recovery [38]. In addition, seeing other women succeed in quitting smoking may also improve one’s self-efficacy of quitting as suggested by the social cognitive theory [39]. It could also help reduce the stigma and shame that is associated with smoking that is known to undermine cessation treatment seeking in some women [40]. It is possible that this will provide women with future gains of increased readiness to ask health care providers and family members for cessation support instead of smoking in secret or denying their smoking [41].

Overall, several participants who described themselves as motivated and ready to quit reported using the behavioral tools in the app with greater engagement. This helped them identify triggers for smoking and come up with alternatives to smoking in these situations. Other participants wished for live support to address challenges, such as chronic medical conditions or mental illness, that come in the way of quitting. This support is most likely to be achieved through interactive counseling by a human or agent. Nonetheless, our results suggest that the MCP-W could be helpful to a subset of women who are willing to use the app in line with their strong commitment to quitting. These findings also indicate that future research should consider stratifying or targeting participants based on motivation and readiness to quit, as well as testing the MCP-W app as part of a blended intervention model (eg, combined with counseling or pharmacotherapy). For example, for women who struggle to engage with the app due to life challenges and low motivation for quitting, a live counseling alternative could be doubly helpful. First, it provides ongoing encouragement and emotional support, and second, it solves problems like how to overcome specific barriers in one’s life, such as having a smoking male partner. Alternately, some women felt that the support could be delivered using a virtual agent such as an AI-based chatbot counselor. Given the advances in the use of AI technologies in health care and the early success of smoking cessation chatbots [35], this is an avenue for scalable low-cost support that should be tested in the future.

### Strengths and Limitations

The strengths of the study include the co-development of gender-specific content for a smoking cessation app by collaborating with patient partners and subject matter experts [42]. In addition, the use of mixed methods ensured that we captured in-depth contextual information about the quantitative findings of the app’s acceptability and feasibility using qualitative data from the interviews [43]. However, most of the participants in this study were treatment-seeking individuals that reported continuing to smoke at the end of a treatment course with the STOP program. This represents a selection bias toward individuals with treatment resistance, which could limit generalizability to women who smoke in the general population [44]. In addition, the study sample size was small and nonparametric tests had to be conducted to explore the acceptability and feasibility. We also acknowledge that the study sample primarily consisted of middle-aged women with higher levels of education, which may limit the generalizability of our findings to younger individuals or those experiencing socioeconomic disadvantage. However, this was an exploratory study, and the attrition rate of participants was low, which allowed us to conduct appropriate statistical analyses.

### Conclusions

The MCP-W app could serve as a valuable tool to support women in their journey to quit smoking. By delivering gender-specific smoking cessation content through a pre-existing gender-neutral platform, the app addresses unique needs. The acceptability and feasibility of the app are closely linked to the motivation and readiness of the woman attempting to quit. Improvements such as incorporating interactive features and offering additional personalized support could enhance its relevance. Feedback from participants highlighted the app’s most helpful features, providing valuable insights. These findings underscore not only the current value of the MCP-W app but also its potential to evolve through continuous improvement, leading to greater effectiveness in women-focused smoking cessation interventions.

## Supplementary material

10.2196/71683 Multimedia Appendix 1 Baseline demographic survey instrument.

10.2196/71683 Multimedia Appendix 2 Surveys for baseline and follow-up.

10.2196/71683 Multimedia Appendix 3 System Usability Scale survey instrument for the follow-up survey.

10.2196/71683 Multimedia Appendix 4 Interview guide.

10.2196/71683 Multimedia Appendix 5 Acceptability of the app based on number of app use days (acceptability is defined by participants who “agreed” or “strongly agreed” with the following statement: “Using the app is likely to help me make changes to my smoking habits”).

## Abbreviations

AI: artificial intelligence
CAMH: Center for Addiction and Mental Health
MCP: My Change Plan
MCP-W: My Change Plan–Women
mhealth: mobile health
REDCap: Research Electronic Data Capture
STOP: Smoking Treatment for Ontario Patients
SUS: System Usability Scale
TFA: Theoretical Framework of Acceptability
