# Supplementary material for: Testing the Acceptability and Feasibility of a Gender-Informed Smoking Cessation mHealth App for Women: Mixed Methods Approach
Source: JMIR Hum Factors. 2025 Sep 25;12:e71683. doi: 10.2196/71683 (PMC12463336; doi:10.2196/71683)
Supplement: Multimedia Appendix 1 [file humanfactors-v12-e71683-s001.docx]

**Appendix 1 - Baseline Demographic Survey Instrument**

1. What is your age in years - ____

|  |  |
| --- | --- |
| 1. Which best describes your current gender identity? (select all that apply)  - Woman - Man - Non-binary (e.g., gender fluid, genderqueer, gender-nonconforming) - Two-spirit - Transgender woman - Gender identity not listed____________ - Do not wish to disclose - Unknown | |

1. What is your marital status?

- Never legally married
- Legally married (and not separated)
- Separated, but still legally married
- Divorced
- Widowed
- Living with a partner but not married (common law)
- Don’t know / prefer not to answer

1. What is the highest level of education you have completed?

- Some primary school
- Primary school
- Some high school
- High school diploma
- Some college
- College diploma
- Some university
- University degree
- Don’t know / prefer not to answer

5. What is your approximate total household income for the past year before income tax deduction (from all sources)?

| □ | No income |
| --- | --- |
| □ | Less than $10,000 |
| □ | $10,001 to $20,000 |
| □ | $20,001 to $40,000 |
| □ | $40,001 to $60,000 |
| □ | $60,001 to $80,000 |
| □ | $80,001 to $100,000 |
| □ | Over $100,000 |
| □ | Don’t know / prefer not to answer |

6. In general, would you say your health is:

- Excellent
- Very good
- Good
- Fair
- Poor

7. The following questions ask about certain chronic health conditions which you may have. We are interested in conditions that have ever been diagnosed by a health professional.

- High blood pressure
- High cholesterol
- Heart disease
- Stroke
- Diabetes
- Chronic bronchitis, emphysema or COPD
- Rheumatoid arthritis
- Chronic pain
- Cancer
- Depression
- Anxiety
- Schizophrenia
- Bipolar disorder
- Substance use disorder (drug addiction other than tobacco or caffeine)
- None of the above
- Don’t know/prefer not to answer

8. In our society, people are often described by their race or racial background. Our race may influence the way we are treated by individuals and institutions, and this may affect our health. Which category(ies) best describes you? Select all that apply:

| □ | First Nations |
| --- | --- |
| □ | Inuit |
| □ | Métis |
| □ | Black (e.g., African, African Canadian, Afro-Caribbean descent) |
| □ | Latin American (Hispanic or Latin American descent) |
| □ | Middle Eastern (e.g., Arab, Persian, West Asian descent [Afghan, Egyptian, Iranian, Kurdish, Lebanese, Turkish, etc.]) |
| □ | East Asian (e.g., Chinese, Japanese, Korean, Taiwanese descent) |
| □ | Southeast Asian (e.g., Cambodian, Filipino, Indonesian, Thai, Vietnamese descent) |
| □ | South Asian (e.g., Bangladeshi, Indian, Indo-Caribbean, Pakistani, Sri Lankan descent) |
| □ | White (e.g., European descent) |
| □ | A race category not described above |
| □ | Do not know |
| □ | Prefer not to answer |
