# Supplementary figures and images for: Testing the Acceptability and Feasibility of a Gender-Informed Smoking Cessation mHealth App for Women: Mixed Methods Approach
Source: JMIR Hum Factors. 2025 Sep 25;12:e71683. doi: 10.2196/71683 (PMC12463336; doi:10.2196/71683)

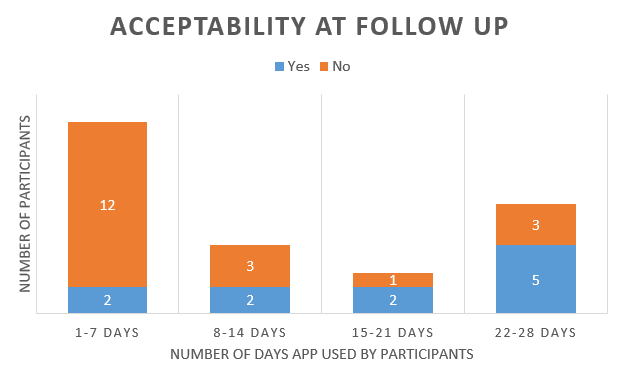

Supplement: Multimedia Appendix 5 [file humanfactors-v12-e71683-s005.png]
